# Ecological and reproductive consequences of endocrine-disrupting chemicals in agricultural systems

**DOI:** 10.1530/RAF-25-0178

**Published:** 2026-03-19

**Authors:** Whitney Payne, Kelsey R Pool

**Affiliations:** School of Agriculture and Environment, The University of Western Australia, Crawley, Western Australia, Australia

**Keywords:** endocrine disruptors, agroecosystems, pesticides, bioaccumulation, livestock reproduction

## Abstract

**Graphical Abstract:**

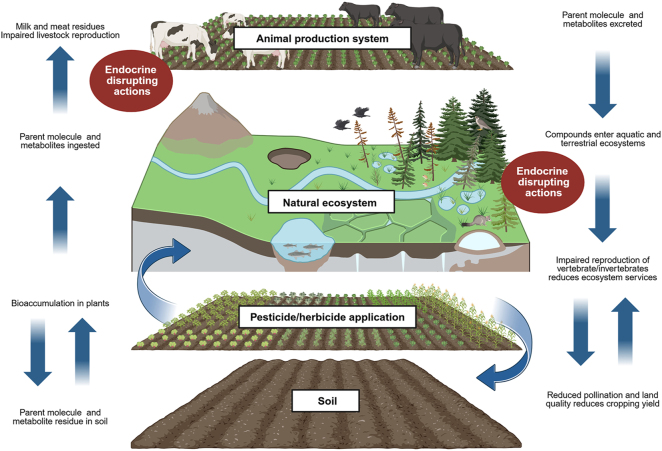

**Abstract:**

Pesticides are a major source of endocrine-disrupting chemicals (EDCs) in agriculture that threaten both reproductive function and ecosystem health. Once applied, pesticides spread beyond their intended treatment area, infiltrating soil and water systems, where non-target species can be exposed. Domestic livestock frequently encounter endocrine-disrupting pesticides in farming environments; however, the effects this exposure has on livestock productivity and reproduction are poorly understood. This review explores the mechanisms through which common insecticides and herbicides disseminate and break down, being the first to consolidate all known metabolites and residues of these chemicals and their persistence and degradation in different matrices. We consider the bioaccumulation and toxicological impacts of pesticides in terrestrial and aquatic ecosystems and how their exposure can impair reproductive function. Organophosphates, carbamates, pyrethroids, neonicotinoids, atrazine, and glyphosate were commonly found to cause reproductive dysfunction in both the male and female through interruptions of hormone function and synthesis and through oxidative stress. Pesticides pose a significant risk to livestock and wildlife through disruptions to reproduction, and given that agricultural resilience and productivity is dependent on the health and function of surrounding ecosystems, the disruptions pesticides cause threaten the ecological balance of agroecosystems. Moving forward, the cumulative impact pesticides and metabolites have on the reproduction of non-target species of livestock, wildlife, and humans must be considered when conducting toxicological assessments of pesticides in addition to their persistence and degradation in agroecosystems. Further research is required to understand how pesticide-induced endocrine disruption affects livestock reproduction.

**Lay summary:**

EDCs are chemicals that interfere with normal hormone production and function. A significant source of EDCs in the environment stems from the use of certain pesticides and herbicides used in food production. Several of the active compounds used for pest management can affect fertility and reproduction in humans, livestock, and wildlife. In agricultural systems, it is unlikely that EDCs remain on farm, as these compounds can move through soil and water, accumulate in plants or animals, and eventually enter the human diet. These pathways link farm management practices to broader environmental and health outcomes. This review explores how EDCs behave and persist within agricultural ecosystems, how they affect reproductive function across species, and how residues can circulate through food chains and adjacent ecosystems. Understanding these interactions helps identify where interventions can reduce exposure and safeguard reproductive health in both agricultural land and natural ecosystems.

## Introduction

Endocrine-disrupting compounds (EDCs) are now recognised as an inescapable feature of modern life. These exogenous chemicals can disrupt the normal function of the endocrine system by mimicking, blocking, or interfering with endogenous hormones. With EDCs derived from a range of natural and synthetic sources (Chintada *et al.* 2025), most living organisms are exposed to EDCs to some degree during both developmental and mature life stages (Chen *et al.* 2025). One of the most critical outcomes of this exposure is the impact on reproductive function (Parent *et al.* 2025, Tzouma *et al.* 2025). Accordingly, EDCs are now suspected to contribute to both male and female reproductive disorders in humans (Tzouma *et al.* 2025), livestock (Boerjan *et al.* 2002, Rhind 2002, Pool *et al.* 2022), and wildlife (Guillette 2001, Carnevali *et al.* 2018).

Agriculture presents a particularly complex and concerning scenario for the ongoing impacts of EDCs. In an agricultural context, EDCs originate from a wide range of sources, including pesticides, plastics, and exogenous hormones used within animal production systems (Rhind 2002). Following their initial release or intake, EDCs and their metabolites rarely remain confined to a single organism or location. Instead, they enter broader ecological networks, moving through terrestrial and aquatic systems, cycling between species, and in many cases returning to impact human health.

While research and regulatory assessments commonly consider direct exposure to pesticides and indirect pathways of exposure through the environment are partially understood, the cumulative burden arising from secondary and tertiary metabolites formed through further degradation and metabolism remains poorly understood. Such exposures are particularly important in agricultural contexts, where farming systems sit at the intersection of animal health, environmental integrity, and food production. Many compounds applied at the farm level can interfere with the soil microbiome, leach into waterways, accumulate in sediments, or persist in the tissues of animals or plants that may enter the human diet (Lakhiar *et al.* 2024). Animal production systems therefore provide a valuable case study for investigating how EDCs can enter diverse food chains and ecosystems from a single source.

This review considers animal production systems not in isolation, but as embedded within larger terrestrial and aquatic ecosystems. In particular, we explore the endocrine-disrupting dynamics of insecticides and herbicides at the scale of direct animal-level exposure on farm and indirect exposure in terrestrial and aquatic agroecosystems, including effects on invertebrates and vertebrates. In light of this information, we consider critical intervention points to mitigate EDC exposure in animal production systems and to understand threshold levels of EDCs in the context of reproduction.

## Pesticide mechanisms and degradation

Major sources of EDCs in agricultural systems are insecticides and herbicides, which are typically regarded as an essential part of pest and weed management. Once present in the environment, pesticides undergo metabolic, microbial, or photolytic degradation, producing metabolites and residues that can differ in toxicity to the parent compound (Cui *et al.* 2023). Often the derivatives of pesticides can also have disruptive effects on physiological and biochemical functions, sometimes with greater potency and persistency than the parent compound (Tang *et al.* 2006). As the breakdown pathway of most pesticide compounds is well elucidated, many studies use known metabolites and residues as biomarkers to quantify the level of environmental contamination in soil, water, and organisms (Vicini *et al.* 2021, Shi *et al.* 2024). Considering the derivatives in addition to the parent compounds broadens our understanding of how pesticides can influence animals and ecological processes across agroecosystems.

### Insecticides

There are five main types of insecticides: organochlorines, organophosphates, carbamates, pyrethroids, and neonicotinoids, which are classified according to their chemical structure or biochemistry. While all these compounds are effective at targeting and reducing pest populations, some are banned due to their potent toxicity to non-target species and their potential to cause ongoing ecological damage. All organochlorine insecticides are banned from use in Australia for this reason, and many organophosphate pesticides have also been banned or are under review. In Europe, neonicotinoids are banned to protect pollinators and other insect populations; however, neonicotinoids are still registered for use in Australia and many other countries (Sgolastra *et al.* 2020). Supplementary Table 1 (see section on Supplementary materials given at the end of the article) lists common insecticides currently registered for use in Australia and their associated metabolites.

Organophosphates were designed as replacements for organochlorine insecticides following their ban in most countries. Organophosphates have a thiophosphate functional group and, when ingested, are bio-transformed into phosphate metabolites, the active form of the insecticide, which have the ability to inhibit acetylcholinesterase activity (Cui *et al.* 2023). By comparison, insecticide carbamates also target and inhibit acetylcholinesterase; however, these effects are reversible due to enzyme-mediated hydrolysis (Colovic *et al.* 2013). By inhibiting the enzyme acetylcholinesterase, acetylcholine accumulates, resulting in hyperstimulation of nicotinic and muscarinic receptors, disrupting neurotransmission, and eventuating to paralysis and death. Similarly, neonicotinoids bind to the nicotinic receptor of insects and cause overstimulation of neurons that will also cause insect death at lethal dosages (Ohno *et al.* 2020). Pyrethroids target insect neurons by preventing the closure of voltage-gated sodium channels, thereby preventing repolarisation and causing overstimulation of neurons (Dong *et al.* 2014). Piperonyl butoxide is a synthetic compound often added to pyrethroid and carbamate insecticides to act as an insecticide synergist (Willoughby *et al.* 2007). Piperonyl butoxide reduces an insect’s ability to break down insecticides by decreasing the metabolic activity of cytochrome P450, a family of enzymes responsible for the metabolism and detoxification of xenobiotics such as insecticides. While these mechanisms effectively control insect pests, their limited species-specificity and potential to cause widespread environmental harm should remain key considerations in insecticide use.

### Herbicides

Herbicides are a diverse group of compounds designed to manage unwanted plant species in both agricultural and metropolitan settings. They can be classified in a variety of ways, such as selectivity (selective vs non-selective), timing of application (preplant, preemergence, and postemergence), application (soil-applied vs foliar-applied), chemical structure (phenoxy acids, triazines, bipyridyliums, etc.), and biochemical mode of action. Typically, herbicides specifically target conserved plant mechanisms, such as photosynthesis and amino acid synthesis, and the auxin signalling pathway, making them theoretically less harmful to animals and humans (Jablonkai 2011). However, mounting evidence suggests that these compounds can impair animal physiological function through endocrine-disrupting activity and oxidative stress (Jabłońska-Trypuć *et al.* 2017). Supplementary Table 2 lists commonly used herbicides registered in Australia and briefly explores their individual mechanisms of action and associated metabolites.

The persistent and non-targeted effects that herbicides have on the environment have recently raised concerns regarding their unintended ecological effects. In 2024, Australia and other countries issued an immediate cancellation of the registration and use of herbicides containing chlorthal dimethyl, also known as dacthal or DCPA (APVMA 2024). The decision was made following a toxicology assessment that identified direct exposure to the compound led to serious health issues primarily in unborn babies, causing low birth weight and impaired brain development (APVMA 2024). The ban reflects a growing recognition of the need for stricter regulation of hazardous pesticides and more comprehensive risk assessments. When conducting environmental and chemical risk assessments, not only should human toxicity be considered but also the environmental fate of pesticides and their degradation, persistency, bioactivity, and impacts on non-target species. Expanding the scope of pesticide toxicological assessments to include these measures ensures that their chemical management protects both human and ecosystem health.

## Exposure to pesticides in agroecosystems

In Australia, repeated treatment of crops with pesticides is standard practice, with farmers reporting that crops such as canola, barley, and wheat are typically sprayed between 5 and 10 times per season. This section will explore how insecticides and herbicides can disseminate into the wider environment through several pathways, exposing non-target organisms across trophic levels and bioaccumulating.

Once applied, pesticides can seep into the soil and disrupt microbial communities by exerting toxic effects, reducing microbial activity, and impairing nutrient cycling pathways (Yasir *et al.* 2025). Within the soil, most pesticides are broken down into their derivatives through microbial and enzymatic activity, as well as abiotic processes such as photodegradation, hydrolysis, and oxidation (Yasir *et al.* 2025). The half-life of insecticides and herbicides in soil varies between compounds with some degrading as quickly as a few hours, while others such as paraquat, atrazine, and bifenthrin have recorded half-lives of over a year (Jones *et al.* 1982, Bromilow 2004, Manzoor & Pervez 2017). The presence of pesticides can compromise soil structure, water retention, and overall soil fertility, which can have ramifications on crop yields in agricultural systems and the stability of the terrestrial ecosystem.

**Figure 1:**
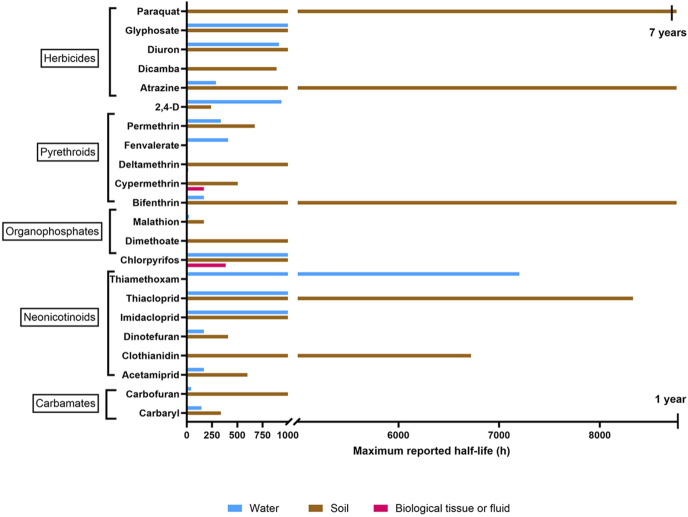
Maximum reported half-life, where known, of common pesticides and herbicides with endocrine-disrupting properties in substrates of water, soil, or biological tissues. References for data presented are listed in Supplementary Table 1.

Pesticides and their derivatives contaminate water systems through surface run-off and groundwater infiltration. Areas adjacent to agricultural land are particularly vulnerable, with concentrations of 294 μg/L of imidacloprid and 12.8 μg/L of dimethoate detected in coastal marine areas intersected by horticultural catchment waterways (Laicher *et al.* 2022). Urban wetlands across Melbourne, Australia, were also found to be contaminated with pesticides, with detected levels of dimethoate (0.002 μg/L), carbaryl (0.004 μg/L), pirimicarb (0.005 μg/L), and atrazine (0.100 μg/L) (Allinson *et al.* 2015). The half-life of insecticides and herbicides in water varies from a few hours to over a year, although most had a reported half-life of a week to a month (Meena *et al.* 2023). The almost ubiquitous presence of these pesticides in aquatic environments can have serious implications for aquatic organisms across all trophic levels. To put the persistence of common pesticides in animals and ecosystems into context, Fig. 1 outlines the maximum reported half-life, where known, of common insecticides and herbicides in substrates of water, soil, or biological tissues.

Plants also serve as a route through which pesticides enter ecosystems. Many plant species can absorb pesticides from direct application or via contaminated water sources and translocate systemic compounds, such as neonicotinoids, some organophosphates, atrazine, glyphosate, and 2,4-D, to specific parts of the plant (Mendes *et al.* 2022). Animals can then be exposed to pesticides by ingesting plants that have absorbed these chemicals or have residues on their surfaces, or by drinking contaminated water.

Once ingested by both vertebrate and invertebrate wildlife, certain pesticides have the capacity to accumulate in biological tissue. Some pesticides are lipophilic and can be stored in fatty tissues rather than being excreted in urine; this can lead to bioaccumulation where the rate of absorption of pesticides is greater than their excretion (Nehul 2025). Pesticides and other endocrine disruptors move through the food chain when predators consume preys that have bioaccumulated compounds in tissues. Their concentrations increase at each trophic level through biomagnification, placing higher-order organisms at a greater risk of exposure to elevated levels of EDCs and experiencing toxic and sub-lethal effects (Tongo *et al.* 2022).

Critical stages of reproduction, such as pregnancy and lactation, involve the mobilisation of fat to supply nutrients to the developing embryo, fetus, or offspring. As a result, bioaccumulated EDCs can be released and transferred to the next generation at a potentially higher concentration than what they would typically be exposed to in their environment (Rhind 2002). Exposure of progeny to EDCs during development can disrupt the tightly regulated events of development and have lasting impacts on growth, physiological, neurological, immune, and reproductive functions (Mallozzi *et al.* 2016). This can occur in wildlife, livestock, and humans alike, highlighting the vulnerability and broad impact EDCs can have across species.

### Impacts of pesticide exposure to livestock

Domestic livestock are often exposed to pesticides in commercial production operations. Depending on the pesticide, exposure may occur via ingestion, for example contaminated water sources or grazing on crop and soil previously treated, via inhalation due to proximity to treated areas, or topically through the skin or mucus membranes (Boerjan *et al.* 2002). Livestock are also able to pass EDCs onto their offspring due to fat mobilisation to support fetal growth and milk production (Rhind 2002). In Argentina, atrazine was present in 89% of tested cow milk samples, with concentrations ranging from 2.51 to 20.97 μg/L (Urseler *et al.* 2022). In addition, the relatively long lifespan of livestock means that these chemicals can accumulate in tissues over periods of multiple years.

Predicting pesticide and residue ingestion of grazing livestock is difficult due to the variability of environmental conditions, production management, and differences in absorption between compounds and individual animals. A recent study provided a framework for the maximum residue levels of pesticides, suggesting ‘safe’ threshold values for most major pesticides ranging between 0.0001 and 0.9 mg/kg dietary intake per day for humans and livestock (Li & Fantke 2022). To place this into context, reported residue levels in livestock feed are far higher, with residues detected in straw (azoxystrobin 0.96 mg/kg), in fodder (cypermethrin 0.143 mg/kg), in oat-based feed (deltamethrin 1.396 mg/kg), in ground corn (piperonyl butoxide 1.505 mg/kg), in durum wheat bran (pirimiphos-methyl 0.562 mg/kg), and in a complete feed (tetramethrin 0.083 mg/kg) (Giugliano *et al.* 2024). Glyphosate residues have also been reported on cereal grains and pulses at concentrations ranging from 0.04 to 5.3 mg/kg (Milesi *et al.* 2021).

The mechanisms by which common EDCs generally disrupt reproductive function are partly elucidated. These mechanisms, in vertebrates, can be broadly distinguished as acting directly on particular reproductive cells or tissues, via downstream effects after systemic disruption, or in a multigenerational manner by epigenetic reprogramming (Akanbi *et al.* 2025, Stanojević & Sollner Dolenc 2025). There are several reviews that discuss the different mechanisms of specific EDCs in mammals at length (Boerjan *et al.* 2002, Rhind 2002, Pool *et al.* 2022, Akanbi *et al.* 2025, Gaillard *et al.* 2025, Stanojević & Sollner Dolenc 2025, Tzouma *et al.* 2025). However, there is a significant gap in current knowledge regarding how pesticides and other EDCs disrupt reproductive function in livestock and further research is required.

### Impacts of pesticides to terrestrial ecosystems

Experimental studies repeatedly confirm the ability of agricultural chemicals to impact the health and reproduction of wildlife. However, there is a lack of studies determining the presence of pesticides, herbicides, and their derivatives *in situ* and clearly linking the presence of these compounds with demonstrated reproductive outcomes in wild populations. Supplementary Table 3 lists studies where these connections have been made, globally. What is clear is that non-target species can encounter chemical residues that are known to have endocrine-disrupting abilities, in concentrations shown experimentally to impair reproduction. The most commonly impacted terrestrial species appear to be birds and insects (Serrão *et al.* 2022, Mohanty 2024). The latter is not surprising given that many pesticides target certain species of insects via conserved biological functions and can accumulate in soil and plants (Oladosu & Flaws 2025). As insects are a critical food source for many animals, this may contribute to the exposure of birds to these compounds. Interestingly, there is a lack of studies looking at the reproductive impact of pesticides on smaller native mammals that also rely on insects for feed.

While detected levels for most pesticide residues in natural ecosystems are typically below 1 mg/kg (Supplementary Table 3), many *in situ* studies lack information around the length and consistency of exposure. By comparison, experimental studies demonstrating sub-lethal effects usually provide a chronic, consistent exposure. Controlled studies therefore are unlikely to mimic the fluctuating dosages realistically encountered by birds, mammals, and above-ground insects within a terrestrial ecosystem. By comparison, however, some pesticides and their metabolites persist in soil (Ore *et al.* 2023), presenting a more consistent source of exposure for ground-dwelling organisms.

Neonicotinoids and organophosphates present a particular issue for below-ground-dwelling organisms. Neonicotinoids are highly water soluble and, consequently, are able to be absorbed by plants and remain as reservoirs in groundwater (Oladosu & Flaws 2025). A recent meta-analysis revealed that exposure to neonicotinoids and organophosphate residues in soil reduces the survival of juveniles, mean number of cocoons, mean cocoons/eggs/cells per animal, mean hatchlings per cocoon, and egg hatchability by more than 80% in orders Oligochaeta and Collembola (Cao *et al.* 2024). Emerging evidence suggests that these impacts may persist beyond the exposed generation, with earthworms exposed to neonicotinoids thiamethoxam and cyantraniliprole demonstrating a reduced reproductive capacity three generations after initial exposure (Martin *et al.* 2024). By comparison, some species, such as predatory mites and springtails, are not impacted even by relatively high neonicotinoid presence (Konestabo *et al.* 2022). Concerningly, this suggests that pesticide residues could rapidly disrupt population ratios and ecosystem balance.

### Impacts of pesticides to aquatic ecosystems

Aquatic ecosystems are particularly vulnerable to insecticide and herbicide residues. Agricultural chemical run-off can accumulate in waterways via transmission of aerosol particles, rain, and groundwater moving through contaminated soils and via animal faeces and urine. Unlike terrestrial ecosystems, aquatic organisms in relatively still water, such as lakes and ponds, are likely to experience chronic or cumulative exposure to chemical residues. This scenario also increases the likelihood of organisms being exposed during developmentally sensitive periods. A recent case study demonstrates the potential for transfer and bioaccumulation of pesticides through trophic levels in a tropical freshwater system (Tongo *et al.* 2022). When bioaccumulation factor was considered as a ratio of pesticide concentrations in biota against in the water, it was found that organisms belonging to the first tropic level, such as phytoplankton, green algae, and macrophytes, exhibit the greatest degree of pesticide bioaccumulation (Tongo *et al.* 2022). This likely represents a secondary dietary exposure pathway for higher-level species, which may perpetuate the effects of pesticides on reproduction beyond initial environmental exposure.

The impacts of pesticide and herbicide residues upon fish and amphibian reproduction are well documented. Pyrethroid cypermethrin in concentrations as low as 1 μg/L are shown to reduce egg hatching rate, alter the length of metamorphosis, and promote morphological deformities in the offspring of moor toads (Greulich & Pflugmacher 2003). Organophosphates, triazines, pyrethroids, carbamates, and organochlorines are also shown to display reproductive toxicity in a number of fish species, disrupting egg hatchability and larvae development (Pašková *et al.* 2011). While the endocrine-disrupting properties of pesticides and herbicides partially account for the observed impairment of reproduction, it is also believed that these outcomes are driven or exacerbated by oxidative stress (Pašková *et al.* 2011).

## Pesticide mechanisms and pathways to reproductive disruption

While little is known about the effects of pesticides on reproductive health of livestock specifically, the endocrine-disrupting pathways of these chemicals have been partially elucidated. Here, we discuss common on-farm EDCs and collate what is known from other species *in vivo* and *in vitro* models to highlight the potential risks to livestock and wildlife in agroecosystems. Although the effects observed in other species and *in vitro* may manifest differently in ruminants, as different metabolite ratios are likely produced during rumination, findings from such studies still provide insights into the mechanisms and potential biological responses.

The typical mechanisms of endocrine disruption include interferences to the oestrogen, progesterone, androgen, and thyroid hormone pathways (Guarnotta *et al.* 2022). Due to the role of these hormones in regulating reproduction, disruptions to these pathways can have adverse effects to animal fertility. In addition, EDCs can induce oxidative stress by increasing the production of reactive oxygen species (ROS) that can damage endocrine and reproductive tissues, which can also impair reproduction (Neier *et al.* 2015). Because these pathways are closely linked to fertility, changes to these pathways induced by EDCs can be interpreted as likely affecting reproductive function. Key factors influencing the extent to which reproductive dysfunction is altered include developmental stage during exposure, concentration, and length of exposure, and whether one or both sexes have been exposed (Rhind 2002).

### Acetylcholinesterase inhibitors (organophosphates and carbamates)

Organophosphates are known to have acute toxicity via inhibition of acetylcholinesterase, an enzyme involved in the hydrolysis of acetylcholine (Martin-Reina *et al.* 2017). While the primary documented side effects of organophosphates are neurological in nature, these compounds still represent a risk of reproductive dysfunction. Acetylcholine has now been found to have many non-neuronal roles in mammals, including the regulation of reproductive processes (Wessler & Kirkpatrick 2017). These roles include sperm motility (Sastry *et al.* 1981), capacitation (Bray *et al.* 2006), the acrosome reaction (Bray *et al.* 2006), and other specific fertilisation processes related to movement of calcium (Bray *et al.* 2006). In the context of female reproduction, acetylcholine regulates oocyte maturation (Kang *et al.* 2011) and, in later stages of embryo development, drives differentiation of the central nervous system (Sam & Bordoni 2023). Outside of inhibition of acetylcholinesterase, organophosphates can also create reproductive dysfunction by disturbing normal redox processes (Ramirez-Vargas *et al.* 2017), altering hormone production (Yang *et al.* 2019, Suwannarin *et al.* 2021, Negi *et al.* 2025), and altering the expression of steroid hormone receptors (Adams *et al.* 2020).

Carbamate-based pesticides also inhibit acetylcholinesterase, thus producing similar reprotoxic effects, although this inhibition is reversible (Sikka & Gurbuz 2005). In addition, these compounds directly promote ROS production, which likely further contributes to the disruption of reproductive processes (Nasrabadi *et al.* 2024). Carbamates interfere with signalling pathways of Nrf2, which plays a critical role in the expression of genes responsible for cellular defence and antioxidant action (Nasrabadi *et al.* 2024).

Organophosphates and carbamates are reported to clinically manifest in a similar manner (Sikka & Gurbuz 2006). Rodent models demonstrate that repeated dietary exposure of carbamates (100 mg/kg) results in significant hormone disruption, with decreases in steroid hormones and inhibin B and an increase in thyroid-stimulating hormone (TSH) (Salem *et al.* 2021).

While the clinical manifestations of pesticide poisoning in livestock are documented for veterinary and agricultural purposes, this information focuses on acute health impacts, rather than more subtle manifestations of reproductive toxicity. As the effects of pesticides on endocrine function and the reproductive system seem to occur after repeated, low-to-moderate exposure, these effects are likely difficult to clinically attribute to pesticide exposure.

Based on data from rodent models, repeated dietary exposure to organophosphate and carbamate parent molecules can impact reproductive function from a dosage of 0.23 mg/kg (Pascotto *et al.* 2015) and 100 mg/kg (Salem *et al.* 2021), respectively. However, *in vitro* ruminant models suggest that endocrine disruption may occur at far lower dosages if reproductive tissues are directly exposed. For example, dosages of carbamate derivative carbaryl at 1 ng/mL were able to disrupt oxytocin signalling, progesterone secretion, and cervical contractions using tissue culture models of bovine cervical tissue (Wrobel *et al.* 2024). Similarly, when caprine testes are directly exposed to 2.5–10 mg/mL of organophosphates and their metabolites in a tissue culture setting, several genes involved in spermatogenesis (genes encoding follicle-stimulating hormone receptor and androgen receptor) are downregulated in response (Mansukhani *et al.* 2024). This suggests that even if the concentration of carbamate or organophosphate derivatives is relatively low in comparison with the ingested dosage, these compounds can impact fertility if they are able to reach reproductive tissues.

*In vitro* work with porcine oocytes and sperm have shown that organophosphates induce ROS production that can lead to cell immaturity and apoptosis, as well as preventing the formation of the blastocyst (Park *et al.* 2024). In livestock, exposure to pesticides targeting acetylcholinesterase could manifest as reduced conception rates due to impaired sperm transport and fertilisation efficiency, irregular oestrous cycles, and early embryonic loss.

### Neonicotinoids

While these compounds are known to cause neurotoxicity via binding to nicotinic acetylcholine receptors (nAChRs), their interaction with reproductive cells and tissues is not well understood. Neonicotinoids and their derivatives appear to reduce reproductive function in both sexes via a combination of endocrine-disrupting properties and promotion of oxidative stress (Oladosu & Flaws 2025). The majority of studies investigating the impacts of neonicotinoids on reproduction have been conducted in small animal models or *in vitro* and primarily model chronic exposure, whereby animals are treated with a consistent dosage of a compound each day (Oladosu & Flaws 2025).

Dietary exposure to acetamiprid at a dosage range of 12.5–35 mg/kg/day in rodents is shown to affect sperm concentration, the percentage of morphologically abnormal sperm, circulating testosterone, and apoptosis in the testes (Arıcan *et al.* 2020). In female mice, intraperitoneal dosages of 10 and 20 mg/kg caused inflammation in the ovaries, along with decreases in secondary and antral follicles (Hassanzadeh *et al.* 2023). These dosages are relatively high, however, which may directly cause inflammation and subsequent oxidative stress, damaging reproductive tissues.

In adult males, similar decreases in sperm production and quality have been reported in rodents exposed to clothianidin (163.4 mg/kg/day and 188.8 mg/kg/day in male and female, respectively), thiacloprid (0.6–6 mg/kg), and imidacloprid (0.06–9 mg/kg) (Oladosu & Flaws 2025). A limitation of these studies is the high, chronic dosages within small animal models, making the potential reprotoxic effects in livestock difficult to extrapolate. Although some neonicotinoids have been investigated at dosages that livestock would encounter on farm during their lifespan and show a detrimental effect on sperm production and function, further research in livestock is needed.

### Pyrethroids

The endocrine-disrupting effects of pyrethroids stem mainly from their ability to mimic thyroid hormones (Leemans *et al.* 2019) and antagonist androgen receptors and inhibit steroid synthesis by modulating oestrogen receptors (Wang *et al.* 2020). Many pyrethroid metabolites have greater endocrine disruptive activity in comparison with their parent molecule (Brander *et al.* 2016). Although known to metabolise relatively rapidly in mammals (Kaneko 2011), the ability of these compounds to accumulate in fats may increase the interaction of parent and derivative molecules with certain tissues and offspring. Outside of direct endocrine-disrupting action, pyrethroids are also thought to cause reproductive impairment via promotion of oxidative stress (Zhang *et al.* 2021).

Animal models support that pyrethroids impact reproductive function. Male rats chronically exposed to dietary cypermethrin (12.5 mg/kg/day) have decreased testosterone, follicle-stimulating hormone (FSH), and luteinising hormone (LH) compared to non-exposed animals, along with a reduced testicular weight, sperm motility, viability, and concentration and increased sperm DNA fragmentation (Alaa-Eldin *et al.* 2017). In the female, pyrethroids fenvalerate, deltamethrin, and cypermethrin have been shown to cause structural changes in the female genital organs, reduce ovulation, promote atresia of follicles, decrease the number of follicular cells, oocytes, and corpora lutea, and induce vesicular atrophy of the endometrial glands (Marettova *et al.* 2017). *In vitro* studies on porcine oocytes have shown that pyrethroids interfere with the maturation of oocytes through inhibiting calcineurin and dysregulating calcium channels, resulting in abnormal meiosis and reduced oocyte quality (Park *et al.* 2024). Importantly, the metabolites produced following the degradation of many pyrethroids have greater oestrogenic and antiandrogenic activity compared to their parent molecules (Tyler *et al.* 2000). This will be discussed in greater detail in a following section.

### Atrazine

Atrazine is a common herbicide for weed control within cropping systems, particularly within Australia and the United States. Unlike many of the other compounds discussed here, atrazine has well-documented endocrine-disrupting properties in wildlife, livestock, and humans, with characterised effects on the reproductive axis (Guimarães-Ervilha *et al.* 2025). A recent meta-analysis outlines that atrazine can disrupt reproductive function by modulating the production of FSH, LH, testosterone, and oestradiol (Guimarães-Ervilha *et al.* 2025). Across studies, in males, it was found that FSH and oestradiol were altered only when atrazine concentrations exceeded 100 mg/kg, while LH and testosterone levels are influenced following exposure concentrations both above and below 100 mg/kg (Guimarães-Ervilha *et al.* 2025). The reported influence in reproductive hormones provides explanation for the observed decrease in sperm function, testicular weight, and morphology, as steroid hormone presence and hypothalamic–pituitary feedback play key roles in the maintenance of spermatogenesis (Guimarães-Ervilha *et al.* 2025). Atrazine is also known to disrupt female reproductive function in mice, and given the reported impacts on hormone production and interference with the hypothalamic–pituitary–gonadal axis, it is perhaps unsurprising that 100 mg/kg atrazine exposure impairs ovarian function, including follicle development and ovulation (Yang *et al.* 2024). In addition, *in vitro* bovine modelling suggests that atrazine also reduces the ability of embryos to successfully implant and divide (Henderson *et al.* 2019).

As with many other pesticides reviewed here, atrazine also increases the production of ROS. Atrazine specifically increases oxidative stress via inhibition of antioxidant enzymes in the hypothalamus and pituitary gland, a pathway partially responsible for disruption of hormone synthesis, secretion, and feedback (Rotimi *et al.* 2024). This likely exacerbates endocrine disruptive effects in both the sexes. Based on the above, atrazine exposure could reduce livestock fertility through altered oestrous cycling, suppressed testicular development, and compromised ovarian follicle maturation.

### Glyphosate

As one of the most widely used herbicides globally (Benbrook 2016), there has been considerable controversy around the role of glyphosate as an endocrine disruptor. However, it has now been shown that glyphosate undeniably modulates hormone synthesis and feedback, with many reports of dysregulated reproductive function in both the male and female (Milesi *et al.* 2021).

Animal models show that glyphosate levels as low as 0.5 mg/kg can reduce reproductive function, whereby mice exposed via drinking water for 4 months had irregular testicular morphology, sperm production, and function (Pham *et al.* 2019). While a comparable study was not performed in the female at this concentration, short-term subcutaneous injection of glyphosate (2 mg/kg/day) in female rats increased oestrogen receptor expression and sensitivity to oestradiol, resulting in increased uterine cell proliferation (Schimpf *et al.* 2018).

There are few studies investigating the impact of glyphosate on livestock reproduction directly. Pre-pubescent female sheep exposed orally to 1 mg/kg/day glyphosate have reduced ovarian reserve, thought to be mediated by the observed reduction in decreased oestrogen receptor-alpha, progesterone receptor, activin receptor II, and bone morphogenetic protein 15 mRNA levels (Alarcón *et al.* 2024). Although an equivalent study does not appear to have been conducted in cattle, *in vitro* research demonstrates that 1 ppm of glyphosate-based herbicide ‘Roundup’ reduces cattle sperm motility and embryo development (Dovolou *et al.* 2024), suggesting that similar impacts on reproduction are possible. In grazing and feedlot settings, chronic glyphosate exposure at subclinical levels may contribute to subfertility through impaired gamete quality and early embryonic development failure. Such effects could manifest as reduced pregnancy rates or extended calving intervals.

### Pesticide metabolites

Increasing evidence supports that the metabolites of pesticides exhibit endocrine-disrupting abilities distinct from the parent compound. Pyrethroid metabolites, 3-PBA and DCCA, exhibit antioestrogenic effects with potencies 100-fold and 1,000-fold, respectively, greater than the parent pyrethroid (Du *et al.* 2010, Sun *et al.* 2014). 3-PBA has also been shown to have antagonistic effects on androgenic and thyroid hormone receptors (Du *et al.* 2010), and antioestrogenic activity has also been observed of an organophosphate metabolite, TCP (Yu *et al.* 2019). The biological activity of pesticide metabolites hints to the potential mechanisms through which they cause endocrine disruption.

Metabolites are often used as biomarkers to indicate an organism’s exposure to pesticides. Despite their ability to be excreted in urine, pesticide metabolites have been detected in blood, adipose tissue, and even breast milk (Yucra *et al.* 2008, Melgarejo *et al.* 2015, Jackson *et al.* 2017). The persistence of 3-PBA and cis- and trans-DCCA metabolites in the body of rats has been reported with half-lives of 4.5, 10, and 14 h in blood and 6.2, 18, and 23 h in the brain, respectively (Starr *et al.* 2014). The relatively long half-life of metabolites in biological tissue prolongs cellular exposure and increases the potential for endocrine-disrupting interactions. Supporting their relatively rapid excretion by mammalian metabolism, there have not been pyrethroid metabolites detected in the meat of farmed animals (Niewiadowska *et al.* 2010). However, metabolites remain detectable in urine 24 h after exposure, in the range of 14–69 pg/mL (Leng *et al.* 1997, Garí *et al.* 2018). This suggests that biologically active metabolites are released into the environment before they can be degraded within the body.

A major limitation of many studies investigating the reproductive impacts of pesticides is the lack of distinction between effects caused by the parent compound and secondary and tertiary metabolites. In humans, associations have been found between the presence of pesticide metabolites in urine and abnormal concentrations of LH, FSH, oestrogen, inhibin B, TSH, and thyroid hormones T3 and T4 (Meeker *et al.* 2006, 2009, Han *et al.* 2008, Hwang *et al.* 2019). The presence of metabolites 3-PBA, DCCA, and TCP in urine are associated with poor sperm function in humans (Meeker *et al.* 2004*a*,*b*, 2009, Ji *et al.* 2011). However, these associations do not distinguish between the effects of the parent compound and the metabolite, despite both compounds having the potential to cause endocrine disruption.

## Conclusion and future perspectives: critical thresholds and intervention points

The routine use of common pesticides in agriculture is no longer an ethically viable option for sustainable food production. Considerable evidence has determined that many classes of insecticides and herbicides can act as endocrine disruptors and have detrimental effects upon reproduction in non-target species. The impact on non-target species not only presents a reduction in the health of agriculturally adjacent ecosystems but is also likely to reduce the productivity of agricultural land.

Moving forward, the registration and use of pesticides and herbicides in food production must consider the cumulative impact on non-target species, as well as the potential to impact reproduction in livestock, wildlife, and humans. In addition, further research is needed on the persistence, pathways for dissemination, and mechanisms on bioaccumulation of pesticides and their derivatives in agroecosystems. The proximity of aquatic ecosystems should be of particular consideration, as well as the ecological role of on-farm non-target invertebrates.

Understanding the mechanisms via which agricultural chemicals impact the reproduction of non-target species is critical. There is currently a significant gap in the literature understanding how pesticides impact livestock reproduction. Livestock studies could also serve as sentinel species for investigating the impacts of pesticide and EDC exposures on non-target species including wildlife, making them highly suitable candidates for future research.

This review explored the downstream effects of pesticide application; however, if we instigated upstream interventions, as in reducing pesticide application, this would reduce the downstream disruptions pesticides cause. This should be considered when designing future pest management strategies, as well as mitigation strategies for ecosystems currently impacted by chemical residues.

Future studies to determine critical thresholds should consider sub-lethal impacts of pesticides on non-target organisms, of which reproduction is an important measure, and should also consider how different pesticides and residues may interact with each other within the body. In addition, concurrent stressors, such as changing climates, nutrition, and pollution, are likely to exacerbate the impacts of chemical exposure. Acceptable thresholds should consider how the interaction of environmental factors may influence the biological activity of residues in certain organisms, with particular focus on oxidative stress.

## Supplementary materials



## Declaration of interest

The authors do not have any actual or perceived conflicts of interest in relation to this review.

## Funding

K Pool was supported by the EHB Lefroy Bequest. W Payne was supported by the WA Agricultural Research Collaboration.

## Author contribution statement

KP and WP collaboratively conceived the scope of the review, wrote the first draft, and edited the review. KP performed final edits and supervision.

## References

[bib1] Adams S, Wiersielis K, Yasrebi A, et al. 2020 Sex- and age-dependent effects of maternal organophosphate flame-retardant exposure on neonatal hypothalamic and hepatic gene expression. Reprod Toxicol 94 65. (10.1016/j.reprotox.2020.04.001) 32360330 PMC7303001

[bib2] Akanbi CA, Rotimi DE, Ojo AB, et al. 2025 Endocrine-disrupting chemicals (EDCs) and epigenetic regulation in embryonic development: mechanisms, impacts, and emerging trends. Toxicol Rep 14 101885. (10.1016/j.toxrep.2024.101885) 40612660 PMC12223400

[bib3] Alaa-Eldin EA, El-Shafei DA & Abouhashem NS 2017 Individual and combined effect of chlorpyrifos and cypermethrin on reproductive system of adult male albino rats. Environ Sci Pollut Control Ser 24 1532–1543. (10.1007/s11356-016-7912-6) 27785720

[bib4] Alarcón R, Alegre AL, Rivera O, et al. 2024 Altered ovarian reserve in Ewe lambs exposed to a glyphosate-based herbicide. Chemosphere 363 142895. (10.1016/j.chemosphere.2024.142895) 39067823

[bib5] Allinson G, Zhang P, Bui A, et al. 2015 Pesticide and trace metal occurrence and aquatic benchmark exceedances in surface waters and sediments of urban wetlands and retention ponds in Melbourne, Australia. Environ Sci Pollut Control Ser 22 10214–10226. (10.1007/s11356-015-4206-3) 25697552

[bib7] APVMA 2024 APVMA cancels all products containing chlorthal dimethyl. (https://www.apvma.gov.au/news-and-publications/news/apvma-cancels-all-products-containing-chlorthal-dimethyl)

[bib8] Arıcan EY, Gökçeoğlu Kayalı D, Ulus Karaca B, et al. 2020 Reproductive effects of subchronic exposure to acetamiprid in male rats. Sci Rep 10 8985. (10.1038/s41598-020-65887-0) 32488017 PMC7265391

[bib9] Benbrook CM 2016 Trends in glyphosate herbicide use in the United States and globally. Environ Sci Eur 28 1–15. (10.1186/s12302-016-0070-0) 27752438 PMC5044953

[bib10] Boerjan ML, Freijnagel S, Rhind SM, et al. 2002 The potential reproductive effects of exposure of domestic ruminants to endocrine disrupting compounds. Anim Sci 74 3–12. (10.1017/s1357729800052164)

[bib11] Brander SM, Gabler MK, Fowler NL, et al. 2016 Pyrethroid pesticides as endocrine disruptors: molecular mechanisms in vertebrates with a focus on fishes. Environ Sci Technol 50 8977–8992. (10.1021/acs.est.6b02253) 27464030

[bib12] Bray C, Son JH & Meizel S 2006 Acetylcholine causes an increase of intracellular calcium in human sperm. Mol Hum Reprod 11 881–889. (10.1093/molehr/gah245) 16421212

[bib13] Bromilow RH 2004 Paraquat and sustainable agriculture. Pest Management Sci Formerly Pestic Sci 60 340–349. (10.1002/ps.823) 15119596

[bib14] Cao Y, Zhao W, Zhang J, et al. 2024 Effects of neonicotinoid residues on non-target soil animals: a case study of meta-analysis. J Hazard Mater 476 135022. (10.1016/j.jhazmat.2024.135022) 38941834

[bib15] Carnevali O, Santangeli S, Forner-Piquer I, et al. 2018 Endocrine-disrupting chemicals in aquatic environment: what are the risks for fish gametes? Fish Physiol Biochem 44 1561–1576. (10.1007/s10695-018-0507-z) 29948447

[bib16] Chen Y, Yuan M, Lin L-Z, et al. 2025 Endocrine disrupting chemicals: sources, mechanisms, and their effects on maternal, fetal, and early childhood health. In Ecological and Human Health Impacts of Contaminated Food and Environments, pp 391–415. CRC Press. (10.1201/9781003492115-25)

[bib17] Chintada V, Veraiah K, Golla N, et al. 2025 Monitoring and Managing Endocrine Disrupter Pesticides (EPDS) for Environmental Sustainability, pp 101–126. Singapore: Springer. (10.1007/978-981-97-7221-6_4)

[bib18] Colovic MB, Krstic DZ, Lazarevic-Pasti TD, et al. 2013 Acetylcholinesterase inhibitors: pharmacology and toxicology. Curr Neuropharmacol 11 315–335. (10.2174/1570159x11311030006) 24179466 PMC3648782

[bib19] Cui J, Wei Y, Jiang J, et al. 2023 Bioaccumulation, metabolism and toxicological effects of chiral insecticide malathion and its metabolites in zebrafish (Danio rerio). Chemosphere 318 137898. (10.1016/j.chemosphere.2023.137898) 36702415

[bib20] Dong K, Du Y, Rinkevich F, et al. 2014 Molecular biology of insect sodium channels and pyrethroid resistance. Insect Biochem Mol Biol 50 1–17. (10.1016/j.ibmb.2014.03.012) 24704279 PMC4484874

[bib21] Dovolou E, Nanas I, Giannoulis T, et al. 2024 The effects of a glyphosate-based herbicide on the bovine gametes during an in vitro embryo production model. Environ Pollut 350 123967. (10.1016/j.envpol.2024.123967) 38631452

[bib22] Du G, Shen O, Sun H, et al. 2010 Assessing hormone receptor activities of pyrethroid insecticides and their metabolites in reporter gene assays. Toxicol Sci 116 58–66. (10.1093/toxsci/kfq120) 20410157

[bib23] Gaillard L, Barouki R, Blanc E, et al. 2025 Per- and polyfluoroalkyl substances as persistent pollutants with metabolic and endocrine-disrupting impacts. Trends Endocrinol Metabol 36 249–261. (10.1016/j.tem.2024.07.021) 39181731

[bib24] Garí M, González-Quinteiro Y, Bravo N, et al. 2018 Analysis of metabolites of organophosphate and pyrethroid pesticides in human urine from urban and agricultural populations (catalonia and Galicia). Sci Total Environ 622-623 526–533. (10.1016/j.scitotenv.2017.11.355) 29220776

[bib25] Giugliano R, Armenio V, Savio V, et al. 2024 Monitoring of non-maximum-residue-level pesticides in animal feed: a study from 2019 to 2023. Toxics 12 680. (10.3390/toxics12090680) 39330608 PMC11435579

[bib26] Greulich K & Pflugmacher S 2003 Differences in susceptibility of various life stages of amphibians to pesticide exposure. Aquat Toxicol 65 329–336. (10.1016/s0166-445x(03)00153-x) 13678851

[bib27] Guarnotta V, Amodei R, Frasca F, et al. 2022 Impact of chemical endocrine disruptors and hormone modulators on the endocrine system. Int J Mol Sci 23 5710. (10.3390/ijms23105710) 35628520 PMC9145289

[bib28] Guillette LJ 2001 Alterations in The Development of The Reproductive and Endocrine Systems of Wildlife Exposed to Endocrine Disrupting Contaminants. In Reproduction, vol 122, pp 857–864. (10.1530/rep.0.1220857) 11732981

[bib29] Guimarães-Ervilha LO, Assis MQ, Bento IPd. S, et al. 2025 Exploring the endocrine-disrupting potential of atrazine for male reproduction: a systematic review and meta-analysis. Reprod Biol 25 100989. (10.1016/j.repbio.2024.100989) 39708576

[bib30] Han Y, Xia Y, Han J, et al. 2008 The relationship of 3-PBA pyrethroids metabolite and male reproductive hormones among non-occupational exposure males. Chemosphere 72 785–790. (10.1016/j.chemosphere.2008.03.058) 18479728

[bib31] Hassanzadeh R, Joursaraei G, Hejazian L, et al. 2023 Evaluation of the protective effect of melatonin on oocyte, embryo and ovarian tissue parameters in female mice exposed to acetamiprid. JBRA Assisted Reprod 27 407. (10.5935/1518-0557.20220068) PMC10712808 37257062

[bib32] Henderson AJ, Finger BJ, Scott AW, et al. 2019 Acute in vitro exposure to environmentally relevant atrazine levels perturbs bovine preimplantation embryo metabolism and cell number. Reprod Toxicol 87 87–96. (10.1016/j.reprotox.2019.05.060) 31129258

[bib33] Hwang M, Lee Y, Choi K, et al. 2019 Urinary 3-phenoxybenzoic acid levels and the association with thyroid hormones in adults: Korean National Environmental Health Survey 2012–2014. Sci Total Environ 696 133920. (10.1016/j.scitotenv.2019.133920) 31446285

[bib34] Jablonkai I 2011 Molecular mechanism of action of herbicides. Herbicides Mechanisms and Mode of Action (IntechOpen) 1–24. (10.5772/31251)

[bib35] Jabłońska-Trypuć A, Wołejko E, Wydro U, et al. 2017 The impact of pesticides on oxidative stress level in human organism and their activity as an endocrine disruptor. J Environ Sci Health B 52 483–494. (10.1080/03601234.2017.1303322) 28541098

[bib36] Jackson E, Shoemaker R, Larian N, et al. 2017 Adipose tissue as a site of toxin accumulation. Compr Physiol 7 1085–1135. (10.1002/cphy.c160038) 28915320 PMC6101675

[bib37] Ji G, Xia Y, Gu A, et al. 2011 Effects of non-occupational environmental exposure to pyrethroids on semen quality and sperm DNA integrity in Chinese men. Reprod Toxicol 31 171–176. (10.1016/j.reprotox.2010.10.005) 20955780

[bib38] Jones T, Kemp W, Stevenson J, et al. 1982 Degradation of Atrazine in Estuarine Water/Sediment Systems and Soils. In J Environ Qual, vol 11, pp 632–638. (10.2134/jeq1982.00472425001100040015x)

[bib39] Kaneko H 2011 Pyrethroids: mammalian metabolism and toxicity. J Agric Food Chem 59 2786–2791. (10.1021/jf102567z) 21133409

[bib40] Kang D, Yun S-Y, Choe C, et al. 2011 Acetylcholine controls mouse oocyte maturation through down-regulation of cAMP. Biol Reprod 85 (Supplement_1) 325. (10.1093/biolreprod/85.s1.325)

[bib41] Konestabo HS, Birkemoe T, Leinaas HP, et al. 2022 Pesticide effects on the abundance of springtails and mites in field mesocosms at an agricultural site. Ecotoxicology 31 1450–1461. (10.1007/s10646-022-02599-3) 36319919 PMC9652236

[bib42] Laicher D, Benkendorff K, White S, et al. 2022 Pesticide occurrence in an agriculturally intensive and ecologically important coastal aquatic system in Australia. Mar Pollut Bull 180 113675. (10.1016/j.marpolbul.2022.113675) 35642798

[bib43] Lakhiar IA, Yan H, Zhang J, et al. 2024 Plastic pollution in agriculture as a threat to food security, the ecosystem, and the environment: an overview. Agronomy 14 548. (10.3390/agronomy14030548)

[bib44] Leemans M, Couderq S, Demeneix B, et al. 2019 Pesticides with potential thyroid hormone-disrupting effects: a review of recent data. Front Endocrinol 10 468622. (10.3389/fendo.2019.00743) PMC6915086 31920955

[bib45] Leng G, Kühn KH & Idel H 1997 Biological monitoring of pyrethroids in blood and pyrethroid metabolites in urine: applications and limitations. Sci Total Environ 199 173–181. (10.1016/s0048-9697(97)05493-4) 9200861

[bib46] Li Z & Fantke P 2022 Framework for defining pesticide maximum residue levels in feed: applications to cattle and sheep. Pest Manag Sci 79 748–759. (10.1002/ps.7241) 36259312 PMC10092036

[bib47] Mallozzi M, Bordi G, Garo C, et al. 2016 The effect of maternal exposure to endocrine disrupting chemicals on fetal and neonatal development: a review on the major concerns. Birth Defects Res C Embryo Today 108 224–242. (10.1002/bdrc.21137) 27653964

[bib48] Mansukhani M, Roy P, Ganguli N, et al. 2024 Organophosphate pesticide chlorpyrifos and its metabolite 3,5,6-trichloropyridinol downregulate the expression of genes essential for spermatogenesis in caprine testes. Pestic Biochem Physiol 204 106065. (10.1016/j.pestbp.2024.106065) 39277380

[bib49] Manzoor F & Pervez M 2017 HPLC analysis to determine the half-life and bioavailability of the termiticides bifenthrin and fipronil in soil. J Econ Entomol 110 2527–2533. (10.1093/jee/tox249) 29040708

[bib50] Marettova E, Maretta M & Legáth J 2017 Effect of pyrethroids on female genital system. Review. Anim Reprod Sci 184 132–138. (10.1016/j.anireprosci.2017.07.007) 28735887

[bib51] Martin-Reina J, Duarte JA, Cerrillos L, et al. 2017 Insecticide reproductive ToxicityProfile: organophosphate, carbamate and pyrethroids. J Toxins 4 7. (10.13188/2328-1723.1000019)

[bib52] Martin WJ, Sibley PK & Prosser RS 2024 Effect of insecticide exposure across multiple generations of the earthworm Eisenia andrei. Environ Toxicol Chem 43 2058–2070. (10.1002/etc.5948) 38980316

[bib53] Meeker JD, Ryan L, Barr DB, et al. 2004a The relationship of urinary metabolites of carbaryl/naphthalene and chlorpyrifos with human semen quality. Environ Health Perspect 112 1665–1670. (10.1289/ehp.7234) 15579410 PMC1253656

[bib54] Meeker JD, Singh NP, Ryan L, et al. 2004b Urinary levels of insecticide metabolites and DNA damage in human sperm. Hum Reprod 19 2573–2580. (10.1093/humrep/deh444) 15333606

[bib55] Meeker JD, Barr DB & Hauser R 2006 Thyroid hormones in relation to urinary metabolites of non-persistent insecticides in men of reproductive age. Reprod Toxicol 22 437–442. (10.1016/j.reprotox.2006.02.005) 16584866

[bib56] Meeker JD, Barr DB & Hauser R 2009 Pyrethroid insecticide metabolites are associated with serum hormone levels in adult men. Reprod Toxicol 27 155–160. (10.1016/j.reprotox.2008.12.012) 19429394 PMC2692246

[bib57] Meena P, Shah PG, Patel KC, et al. 2023 Dissipation behaviour of bifenthrin in water at different pH levels under laboratory conditions. Int J Environ Anal Chem 103 8756–8764. (10.1080/03067319.2021.1996568)

[bib58] Melgarejo M, Mendiola J, Koch HM, et al. 2015 Associations between urinary organophosphate pesticide metabolite levels and reproductive parameters in men from an infertility clinic. Environ Res 137 292–298. (10.1016/j.envres.2015.01.004) 25601731

[bib59] Mendes KF, Mielke KC, D’Antonino L, et al. 2022 Retention, absorption, translocation, and metabolism of herbicides in plants. In Applied Weed and Herbicide Science, pp 157–186. Springer. (10.1007/978-3-031-01938-8_5)

[bib60] Milesi MM, Lorenz V, Durando M, et al. 2021 Glyphosate herbicide: reproductive outcomes and multigenerational effects. Front Endocrinol 12 672532. (10.3389/fendo.2021.672532) PMC8293380 34305812

[bib61] Mohanty B 2024 Pesticides exposure and compromised fitness in wild birds: focusing on the reproductive endocrine disruption. Pestic Biochem Physiol 199 105800. (10.1016/j.pestbp.2024.105800) 38458691

[bib62] Nasrabadi M, Nazarian M, Darroudi M, et al. 2024 Carbamate compounds induced toxic effects by affecting Nrf2 signaling pathways. Toxicol Rep 12 148–157. (10.1016/j.toxrep.2023.12.004) 38304697 PMC10831123

[bib63] Negi CK, Gadara D, Bajard L, et al. 2025 2-Ethylhexyl diphenyl phosphate affects steroidogenesis and lipidome profile in human adrenal (H295R) cells. Chem Res Toxicol 38 733–744. (10.1021/acs.chemrestox.5c00030) 40178524 PMC12015954

[bib64] Nehul JN 2025 Environmental impact of pesticides: toxicity, bioaccumulation and alternatives. Environ Rep 7 14–21. (10.51470/er.2025.7.2.14)

[bib65] Neier K, Marchlewicz EH, Dolinoy DC, et al. 2015 Assessing human health risk to endocrine disrupting chemicals: a focus on prenatal exposures and oxidative stress. Endocr Disruptors 3 e1069916. (10.1080/23273747.2015.1069916) PMC4876868 27231701

[bib66] Niewiadowska A, Kiljanek T, Semeniuk S, et al. 2010 Determination of pyrethroid residues in meat by gas chromatography with electron capture detection. Bull Vet Inst Pulawy 54 595–599.

[bib67] Ohno S, Ikenaka Y, Onaru K, et al. 2020 Quantitative elucidation of maternal-to-fetal transfer of neonicotinoid pesticide clothianidin and its metabolites in mice. Toxicol Lett 322 32–38. (10.1016/j.toxlet.2020.01.003) 31923464

[bib68] Oladosu JI & Flaws JA 2025 The impact of neonicotinoid pesticides on reproductive health. Toxicol Sci 203 131–146. (10.1093/toxsci/kfae138) 39460954 PMC11775419

[bib69] Ore OT, Adeola AO, Bayode AA, et al. 2023 Organophosphate pesticide residues in environmental and biological matrices: occurrence, distribution and potential remedial approaches. Environ Chem Ecotoxicol 5 9–23. (10.1016/j.enceco.2022.10.004)

[bib70] Parent AS, Damdimopoulou P, Johansson HKL, et al. 2025 Endocrine-disrupting chemicals and female reproductive health: a growing concern. Nat Rev Endocrinol 21 593–607. (10.1038/s41574-025-01131-x) 40404936

[bib71] Park J, Lee H, Kweon J, et al. 2024 Mechanisms of female reproductive toxicity in pigs induced by exposure to environmental pollutants. Mol Cells 47 100065. (10.1016/j.mocell.2024.100065) 38679414 PMC11143778

[bib72] Pascotto VM, Guerra MT, Franci JAA, et al. 2015 Effects of a mixture of pesticides on the adult female reproductive system of Sprague-Dawley, Wistar, and Lewis rats. J Toxicol Environ Health A 78 602–616. (10.1080/15287394.2015.1010467) 25965195

[bib73] Pašková V, Hilscherová K & Bláha L 2011 Teratogenicity and embryotoxicity in aquatic organisms after pesticide exposure and the role of oxidative stress. Rev Environ Contam Toxicol 211 25–61. (10.1007/978-1-4419-8011-3_2) 21287390

[bib74] Pham TH, Derian L, Kervarrec C, et al. 2019 Perinatal exposure to glyphosate and a glyphosate-based herbicide affect spermatogenesis in mice. Toxicol Sci 169 260–271. (10.1093/toxsci/kfz039) 30785197

[bib75] Pool KR, Chazal F, Smith JT, et al. 2022 Estrogenic pastures: a source of endocrine disruption in sheep reproduction. Front Endocrinol 13 880861. (10.3389/fendo.2022.880861) PMC9097266 35574027

[bib76] Ramirez-Vargas MA, Huerta-Beristain G, Guzman-Guzman IP, et al. 2017 Methamidophos induces cytotoxicity and oxidative stress in human peripheral blood mononuclear cells. Environ Toxicol 32 147–155. (10.1002/tox.22220) 26589457

[bib77] Rhind SM 2002 Endocrine disrupting compounds and farm animals: their properties, actions and routes of exposure. Domest Anim Endocrinol 23 179–187. (10.1016/s0739-7240(02)00155-8) 12142236

[bib78] Rotimi DE, Ojo OA & Adeyemi OS 2024 Atrazine exposure caused oxidative stress in male rats and inhibited brain-pituitary-testicular functions. J Biochem Mol Toxicol 38 e23579. (10.1002/jbt.23579) 37926918

[bib79] Salem MA, Ismail RS, Zaki HF, et al. 2021 L-carnitine extenuates endocrine disruption, inflammatory burst and oxidative stress in carbendazim-challenged male rats via upregulation of testicular StAR and FABP9, and downregulation of P38-MAPK pathways. Toxicology 457 152808. (10.1016/j.tox.2021.152808) 33965443

[bib80] Sam C & Bordoni B 2023 Physiology, acetylcholine. In StatPearls. Treasure Island, FL, USA: StatPearls Publishing. (https://www.ncbi.nlm.nih.gov/books/NBK557825/) 32491757

[bib81] Sastry BVR, Janson VE & Chaturvedi AK 1981 Inhibition of human sperm motility by inhibitors of choline acetyltransferase. J Pharmacol Exp Therapeut 216 378–384. (10.1016/s0022-3565(25)32430-4) 7463354

[bib82] Schimpf MG, Milesi MM, Luque EH, et al. 2018 Glyphosate-based herbicide enhances the uterine sensitivity to estradiol in rats. J Endocrinol 239 197–213. (10.1530/joe-18-0207) 30121576

[bib83] Serrão JE, Plata-Rueda A, Martínez LC, et al. 2022 Side-effects of pesticides on non-target insects in agriculture: a mini-review. Sci Nat 109 1–11. (10.1007/s00114-022-01788-8) 35138481

[bib84] Sgolastra F, Medrzycki P, Bortolotti L, et al. 2020 Bees and pesticide regulation: lessons from the neonicotinoid experience. Biol Conserv 241 108356. (10.1016/j.biocon.2019.108356)

[bib85] Shi T, Zhang Q, Chen X, et al. 2024 Overview of deltamethrin residues and toxic effects in the global environment. Environ Geochem Health 46 271. (10.1007/s10653-024-02043-x) 38954040

[bib107] Sikka SC & Gurbuz N 2005 Reproductive Toxicity of Organophosphate and Carbamate Pesticides. In: Toxicology of Organophosphate and Carbamate Compounds. Academic Press pp 447–462. (10.1016/B978-012088523-7/50033-8)

[bib86] Stanojević M & Sollner Dolenc M 2025 Mechanisms of bisphenol A and its analogs as endocrine disruptors via nuclear receptors and related signaling pathways. Arch Toxicol 99 2397–2417. (10.1007/s00204-025-04025-z) 40116906 PMC12185661

[bib87] Starr JM, Graham SE, Ross DG, et al. 2014 Environmentally relevant mixing ratios in cumulative assessments: a study of the kinetics of pyrethroids and their ester cleavage metabolites in blood and brain; and the effect of a pyrethroid mixture on the motor activity of rats. Toxicology 320 15–24. (10.1016/j.tox.2014.02.016) 24631210

[bib88] Sun H, Chen W, Xu X, et al. 2014 Pyrethroid and their metabolite, 3-phenoxybenzoic acid showed similar (anti) estrogenic activity in human and rat estrogen receptor α-mediated reporter gene assays. Environ Toxicol Pharmacol 37 371–377. (10.1016/j.etap.2013.11.031) 24388911

[bib89] Suwannarin N, Prapamontol T, Isobe T, et al. 2021 Exposure to organophosphate and neonicotinoid insecticides and its association with steroid hormones among male reproductive-age farmworkers in Northern Thailand. Int J Environ Res Publ Health 18 5599. (10.3390/ijerph18115599) PMC8197278 34073889

[bib90] Tang J, Rose RL & Chambers JE 2006 Metabolism of organophosphorus and carbamate pesticides. In Toxicology of Organophosphate & Carbamate Compounds, pp 127–143. Academic Press. (10.1016/B978-012088523-7/50011-9)

[bib91] Tongo I, Onokpasa A, Emerure F, et al. 2022 Levels, bioaccumulation and biomagnification of pesticide residues in a tropical freshwater food web. Int J Environ Sci Technol 19 1467–1482. (10.1007/s13762-021-03212-6)

[bib92] Tyler CR, Beresford N, Van Der Woning M, et al. 2000 Metabolism and environmental degradation of pyrethroid insecticides produce compounds with endocrine activities. Environ Toxicol Chem 19 801–809. (10.1897/1551-5028(2000)019<0801:maedop>2.3.co;2)

[bib93] Tzouma Z, Dourou P, Diamanti A, et al. 2025 Associations between endocrine-disrupting chemical exposure and fertility outcomes: a decade of human epidemiological evidence. Life 15 993. (10.3390/life15070993) 40724496 PMC12299029

[bib94] Urseler N, Bachetti R, Biolé F, et al. 2022 Atrazine pollution in groundwater and raw bovine milk: water quality, bioaccumulation and human risk assessment. Sci Total Environ 852 158498. (10.1016/j.scitotenv.2022.158498) 36063942

[bib96] Vicini JL, Jensen PK, Young BM, et al. 2021 Residues of glyphosate in food and dietary exposure. Compr Rev Food Sci Food Saf 20 5226–5257. (10.1111/1541-4337.12822) 34397175

[bib97] Wang Q, Shen JY, Zhang R, et al. 2020 Effects and mechanisms of pyrethroids on male reproductive system. Toxicology 438 152460. (10.1016/j.tox.2020.152460) 32278050

[bib98] Wessler IK & Kirkpatrick CJ 2017 Non-neuronal acetylcholine involved in reproduction in mammals and honeybees. J Neurochem 142 144–150. (10.1111/jnc.13953) 28072454

[bib99] Willoughby L, Batterham P & Daborn PJ 2007 Piperonyl butoxide induces the expression of cytochrome P450 and glutathione s‐transferase genes in drosophila melanogaster. Pest Management Sci Formerly Pestic Sci 63 803–808. (10.1002/ps.1391) 17514638

[bib100] Wrobel MH, Mlynarczuk J & Rekawiecki R 2024 Effects of commonly used carbamates (carbaryl and thiram) on the regulatory, secretory and motor functions of bovine cervixes in vitro. Theriogenology 218 183–192. (10.1016/j.theriogenology.2024.01.030) 38330862

[bib101] Yang F, Li J, Pang G, et al. 2019 Effects of diethyl phosphate, a non-specific metabolite of organophosphorus pesticides, on serum lipid, hormones, inflammation, and gut microbiota. Molecules 24 2003. (10.3390/molecules24102003) 31137755 PMC6572208

[bib102] Yang YF, Cheng SY, Wang YL, et al. 2024 Accumulated inflammation and fibrosis participate in atrazine induced ovary toxicity in mice. Environ Pollut 360 124672. (10.1016/j.envpol.2024.124672) 39103034

[bib103] Yasir M, Hossain A & Pratap-Singh A 2025 Pesticide degradation: impacts on soil fertility and nutrient cycling. Environments 12 272. (10.3390/environments12080272)

[bib104] Yu C, Wang C, Lu Z, et al. 2019 The endocrine-disrupting potential of four chlorophenols by in vitro and in silico assay. Chemosphere 218 941–947. (10.1016/j.chemosphere.2018.11.199) 30609499

[bib105] Yucra S, Gasco M, Rubio J, et al. 2008 Semen quality in Peruvian pesticide applicators: association between urinary organophosphate metabolites and semen parameters. Environ Health 7 1–10. (10.1186/1476-069x-7-59) 19014632 PMC2588569

[bib106] Zhang X, Zhang T, Ren X, et al. 2021 Pyrethroids toxicity to Male reproductive system and offspring as a function of oxidative stress induction: rodent studies. Front Endocrinol 12 656106. (10.3389/fendo.2021.656106) PMC8190395 34122335

