# Supplementary material for: Ecological and reproductive consequences of endocrine-disrupting chemicals in agricultural systems
Source: Reprod Fertil. 2026 Mar 19;7(1):RAF250178. doi: 10.1530/RAF-25-0178 (PMC13011801; doi:10.1530/RAF-25-0178)
Supplement: Supplementary file 1 [file supplementary_materials.pdf]

**Supplementary table 1.** Common insecticides used in first world cropping systems, their biological mode of action to manage weed species, and subsequent metabolites of the parent compounds.

| Insecticide class | Biological mechanism of action | Compound | Metabolites | Reference | Degradation half-life (parent compound) | | |
|---|---|---|---|---|---|---|---|
| | | | | | Matrix | Time | Reference |
| Organophosphate | Acetylcholinesterase inhibitor | Malathion | Malaoxon<br>Malaoxon monocarboxylic acid (DMA)<br>Malaoxon dicarboxylic acid (DCA)<br>Dimethyl phosphate (DMP)<br>Dimethyl thiophosphate (DMTP)<br>Dimethyl dithiophosphate (DMDTP)<br>Dimethyl mercapto phosphate succinate<br>Diethyl mercapto succinate<br>Diethyl succinate<br>Succinate | (Singh et al., 2014) | Water | 0.5-3 d | (Cotham & Bidleman, 1989; Cui et al., 2023; Wolfe et al., 1977) |
| | | | | | Soil | Few hours to 1 w | (Singh et al., 2014) |
| | | | | | Biological tissue/fluid | 3 h (zebrafish) | (Cui et al., 2023) |
| | | Chlorpyrifos | Chlorpyrifos-oxon<br>3,5,6-trichloro-pyridinol (TCP)<br>Diethyl phosphorothioate (DETP) | (Bose et al., 2021) | Water | 35-78 d | (Huang et al., 2021) |

| | | | | | | | |
|---|---|---|---|---|---|---|---|
| | | | 3,5,6-trichloro-2-methypyridine (TMP)<br>3,5,6-trichloro-2-methoxypyridine (TCMP)<br>Chlorodihydro-2-pyridone<br>Tetrahydro-2-pyridone<br>Maleimide semialdehyde | | Soil | 10-120 d | (Huang et al., 2021) |
| | | | | | Biological tissue/fluid | 55 s (human blood) | (Bouchard et al., 2005) |
| | | Dimethoate | Omethoate<br>Dimethyl dithiophosphate (DMDTP)<br>Dimethyl phosphate (DMP)<br>Dimethoate carboxylic acid (DMCA)<br>Dimethyl thiophosphate (DMTP)<br>O, Desmethyldithiophosphate | (Ahmad et al., 2022; Nallani et al., 2023) | Water | 20 d | (Anićijević et al., 2022) |
| | | | | | Soil | >200 d | (Ahmad et al., 2022) |
| | | | | | Biological tissue/fluid | 4-6 h (human liver)<br>1 h (rat liver) | (Nallani et al., 2023) |
| Carbamate | Reversible acetylcholinesterase inhibitor | Carbofuran | 3-hydroxy-carbofuran<br>3-keto carbofuran<br>3-hydroxycarbofuran phenol<br>3-keto-carbofuran-7-phenol | (Petropoulou et al., 2006) | Water | 1-3 d | (de Melo Plese et al., 2005) |
| | | | | | Soil | 4-15 d | (Parkin & Shelton, 1992) |
| | | | | | Biological tissue/fluid | 3 h – 2 d (human) | (Petropoulou et al., 2006) |
| | | Carbaryl | 1-naphthol | (Petropoulou et al., 2006; | Water | 1-6 d | (Sunaryani & |

| | | | | | | | |
|---|---|---|---|---|---|---|---|
| | | | 5-hydroxycarbaryl<br>4-hydroxycarbaryl<br>Carbaryl methylol | Tang et al., 2002) | | | Rosmalina, 2021) |
| | | | | | Soil | 5-14 d | (Sunaryani & Rosmalina, 2021) |
| | | | | | Biological tissue/fluid | Unknown | |
| | | Pirimicarb | Desmethyl pirimicarb<br>Desmethyl-formamido pirimicarb | (Kızılarslan et al., 2025) | Water | Unknown | |
| | | | | | Soil | Unknown | |
| | | | | | Biological tissue/fluid | Unknown | |
| Pyrethroid | Prevent closure of voltage-gated sodium channels | Cypermethrin | 3-phenoxybenzoic acid (3-PBA)<br>*cis*- or *trans*-3-(2,2 dichlorovinyl)-2,2-dimethyl-(1-cyclopropane) carboxylic acid (*cis*- and *trans*-DCCA)<br>3-phenoxybenzaldehyde<br>3-(4-hydroxy-3-phenoxy)benzyl alcohol<br>3-(4-hydroxy-3-phenoxy)benzoic acid<br>N-3-(phenoxybenzoyl)glycine | (McCarthy et al., 2006; Willemin et al., 2015) | Water | 1-3 w | (Xie & Zhou, 2008) |
| | | | | | Soil | 2 h | (Liu et al., 2007) |
| | | | | | Biological tissue/fluid | Unknown | |
| | | Permethrin | 3-PBA<br>*cis*- and *trans*-DCCA<br>3-phenoxybenzyl alcohol | (McCarthy et al., 2006; Willemin et al., 2015) | Water | 14 d | (Schimmel et al., 1983) |
| | | | | | Soil | <4 w | (Sharom & Solomon, 1981) |

| | | | | | | | |
|---|---|---|---|---|---|---|---|
| | | | 3-phenoxybenzaldehyde | | Biological tissue/fluid | Unknown | |
| | | Fenvalerate | 3-PBA<br>*cis-* and *trans*-DCCA | (Sun et al., 2014) | Water | 8-17 d | (Cotham & Bidleman, 1989; Schimmel et al., 1983) |
| | | | | | Soil | 5-6 w | (Hill, 1981) |
| | | | | | Biological tissue/fluid | Unknown | |
| | | Bifenthrin | 3-PBA<br>*cis-* and *trans*-DCCA<br>3-phenoxybenzyl alcohol<br>3-phenoxybenzaldehyde<br>4'-hydroxy-bifenthrin<br>Biphenyl acid<br>Biphenyl alcohol<br>3,2-(chloro-3,3,3-trifluoro-1-propenyl)-2,2-dimethylcyclopropane carboxylate | (Yang et al., 2018) | Water | 1 w | (Meena et al., 2023) |
| | | | | | Soil | 1 y | (Manzoor & Pervez, 2017) |
| | | | | | Biological tissue/fluid | 11.1 h and 13.4 h (rat brain and plasma, respective) | (Gammon et al., 2015) |
| | | Deltamethrin | 3-PBA<br>cis-3-(2,2-dibromovinyl)-2,2dimethylcyclopropane carboxylic acid (cis- | (Shi et al., 2024) | Water | 2-4 h | (Muir et al., 1985) |

| | | | | | | | |
|---|---|---|---|---|---|---|---|
| | | | DBCA)<br>2’-OH-deltamethrin<br>4’-OH-deltamethrin<br>2’-OH-PBA<br>4’-OH-PBA<br>3-hydroxybenzoic acid | | Soil | 3-8 w | (Ismail et al., 2015) |
| | | | | | Biological tissue/fluid | 10-13 h (plasma and urine) | (Siddiqui & Desai, 2020) |
| Neonicotinoid | Binds nicotinic receptor | Thiamethoxam | Desmethyl-thiamethoxam<br>Clothianidin<br>See clothianidin for further break down of metabolites | (Ford & Casida, 2006) | Water | >1 y | (Maienfisch et al., 2001) |
| | | | | | Soil | 7-353 d | (Goulson, 2013) |
| | | | | | Biological tissue/fluid | 2.7 h (*Eremias argus*) | (Wang et al., 2018) |
| | | Imidacloprid | 5-hydroxyimidacloprid<br>6-chloronicotinic acid<br>Olefin-imidacloprid<br>Imidacloprid-urea<br>Imidacloprid guanidine | (Schoning & Schmuck, 2003; Suchail et al., 2004) | Water | 3 d | (Fouad & Abdel-Raheem, 2024) |
| | | | | | Soil | 36-89 d | (Fouad & Abdel-Raheem, 2024) |
| | | | | | Biological tissue/fluid | 68 h (rainbow trout) | (Frew et al., 2018) |
| | | Clothianidin | Desmethyl-clothianidin | (Ohno et al., 2020) | Water | 1-4 h | (Kah et al., 2018) |

| | | | | | | | |
|---|---|---|---|---|---|---|---|
| | | | Desmethyl-desnitro-clothianidin<br>1-methyl-3-nitroguanidine<br>desnitro-clothianidin<br>clothianidin-urea | | Soil | 90-280 d | (Li et al., 2018) |
| | | | | | Biological tissue/fluid | Unknown | |
| | | Dinotefuran | 1-methyl-2-nitroguanidine (MNG)<br>1-methyl-3-(tetrahydro-3-furylmethyl) urea (UF)<br>1-methyl-3-tetrahydro-3-furylmethyl guanidine (DN) | (Li et al., 2017) | Water | 7 d | (Soares et al., 2023) |
| | | | | | Soil | 8-17 d | (Li et al., 2020) |
| | | | | | Biological tissue/fluid | Unknown | |
| | | Thiacloprid | Thiacloprid amide<br>6-chloronicotinic acid (6-CNA)<br>4-hydroxy thiacloprid<br>4-ketone thiacloprid imine | (Pan et al., 2025; Zhao et al., 2025) | Water | 173 d | (Chen et al., 2021) |
| | | | | | Soil | 40-347 d | (Chen et al., 2021) |
| | | | | | Biological tissue/fluid | Unknown | |
| | | Acetamiprid | N-desmethyl-acetamiprid<br>6-CNA | (Phogat et al., 2022) | Water | 7 d | (Pitam et al., 2013) |
| | | | | | Soil | 25 d | (Gupta et al., 2008) |
| | | | | | Biological tissue/fluid | 0.5 h | (Brunet et al., 2005) |

**Supplementary table 2.** Common herbicides used in first world cropping systems, their biological mode of action to manage weed species, and subsequent metabolites of the parent compounds.

| Compound | Biological mechanism of action | Metabolites | Reference | Matrix | Degradation Time | Reference |
|---|---|---|---|---|---|---|
| Atrazine | Photosystem II inhibitor<br><br>Binds to the D1 protein, thereby blocking quinone from binding to the site and disrupting the electron transport chain. This causes electrons to accumulate, resulting in high levels of oxidation that kill the plant. | Desethylatrazine<br>Desisopropylatrazine<br>Diamino-s-chlorotriazine<br>1-hydroxyisopropylatrazine<br>2-hydroxyatrazine<br>Cyanuric acid | (Joo et al., 2010; Laws et al., 2009) | Water | 3-12 d | (Jones et al., 1982) |
| | | | | Soil | 330-385 d | (Jones et al., 1982) |
| | | | | Biological tissue/fluid | Unknown | |
| Glyphosate | EPSPS inhibitor<br><br>Prevents the synthesis of aromatic amino acids by inhibiting the enzyme 5-enolpyruvylshikimate-3-phosphate synthase (EPSPS). | Aminomethylphosphonic acid (AMPA)<br>Glyoxylic acid<br>Sarcosine<br>Glycine | (Vicini et al., 2021) | Water | 49-70 d | (Mercurio et al., 2014) |
| | | | | Soil | 5-174 d | (Mercurio et al., 2014) |
| | | | | Biological tissue/fluid | 10 h (urine) | (Connolly et al., 2019) |
| 2,4-D<br>2,4- | Synthetic auxin | 2,4-dichlorophenol<br>4-hydroxy-2,5- | (Feung et al., 1975; | Water | 13-39 d | (Boivin et al., 2005) |

| | | | | | | |
|---|---|---|---|---|---|---|
| dichlorophenoxyacetic acid | Causes uncontrolled, unsustainable growth resulting in senescence and death. | dichlorophenoxyacetic acid<br>4-hydroxy-2,3-dichloropenhoxyacetic acid<br>4-chlorophenoxyacetic acid | Van Ravenzwaay et al., 2003) | Soil | 5-10 d | (Wafa et al., 2011) |
| | | | | Biological tissue/fluid | Unknown | |
| Dicamba | Synthetic auxin<br><br>Causes uncontrolled, unsustainable growth resulting in senescence and death. | 3,6-dichlorosalicylic acid (DCSA)<br>5-hydroxy-3,6-dichloro-*o*-anisic acid | (Chang & Vanden Born, 1971) | Water | Unknown | |
| | | | | Soil | 37 d | (Roy et al., 2001) |
| | | | | Biological tissue/fluid | Unknown | |
| Paraquat | Photosystem I inhibitor<br><br>Oxidises ferredoxins that are responsible for mediating the electron transport chain. These “stolen” electrons then generate high levels of reactive oxygen species (ROS) that kill the plant. | 1-methyl-4-carboxypyridium ion | (Funderburk & Bozarth, 1967) | Water | Unknown | |
| | | | | Soil | 1-7 y | (Bromilow, 2004) |
| | | | | Biological tissue/fluid | 1-3 month (mouse brain) | (Prasad et al., 2009) |
| Diuron | Photosystem II inhibitor<br><br>Binds to the D1 protein, thereby blocking quinone from binding to the site and disrupting the electron transport chain. This causes electrons to accumulate, resulting in high levels of oxidation that kill the plant. | 3,4-dichloroaniline (DCA)<br>3-(3,4-dichlorophenyl)urea<br>3-(3,4-dichlorophenyl)-1-methyl urea<br>3,4-dichlorophenol<br>3-(3-chloro-4-hydroxyphenyl)-dimethylurea<br>3-(4-chloro-3-hydroxyphenyl)-dimethylurea | (Giacomazzi & Cochet, 2004) | Water | 9-38 d | (Sakugawa et al., 2010) |
| | | | | Soil | 22-50 d | (Muhamad et al., 2013) |
| | | | | Biological tissue/fluid | Unknown | |

**Supplementary table 3.** Published studies demonstrating the presence of pesticides, herbicides and their derivatives in situ in natural ecosystems, where reproductive outcomes in wild animal populations were measured.

| Compound/s detected and dosage | Species exposed via secondary exposure | Method of secondary exposure | Effect on reproduction | Reference |
|---|---|---|---|---|
| Reported as concentrations in cattle faeces. Pyrethroids: Cypermethrin (0.0326 mg/kg), deltamethrin (0.116 mg/kg), permethrin-cis (0.144 mg/kg), permethrin-trans (0.258 mg/kg), tefluthrin (0.007).<br><br>Neonicotinoids: imidacloprid (0.009 mg/kg).<br><br>Organophosphates: pirimiphos-methyl (0.221 mg/kg).<br><br>Carbamates: chlorpropham (0.322 mg/kg).<br><br>Phenoxy acids: 2, 4-D (0.018 mg/kg), MCPA (0.017 mg/kg), mecoprop (0.055 mg/kg).<br><br>Triazines: Terbuthylazine (0.026 mg/kg) and it's metabolite Terbuthylazine-desethyl (0.053 mg/kg). | Unspecified insect species from families Staphylinidae and Scarabaeidae (Order Coleoptera) and unspecified species from Order Diptera. | Residues present in cattle faeces, which are a food source for the observed insect species. | No effect on Diptera. Reduced numbers of both larvae and adults of Coleoptera. | (Bruinenberg et al., 2023) |
| Reported as concentrations detected in | Great Tit (*Parus* | Animal hair/fur | Number of dead offspring | (Tassin de |

| | | | | |
|---|---|---|---|---|
| nesting material.<br>Phenylpyrazoles (fiproles): Fipronil (0.118 mg/kg), fipronil sulfone (0.0879 mg/kg), fipronil sulfide (0.00334 mg/kg)<br><br>Neonicotinoids: Imidacloprid (0.358 mg/kg), desnitro-imidacloprid (0.0125 mg/kg), imidacloprid-olefin (0.0115 mg/kg), acetamiprid (0.00147 mg/kg), desmethyl-acetamiprid (0.00006 mg/kg), thiacloprid (0.00044 mg/kg), thiamethoxam (0.00034 mg/kg), dinotefuran (7.198 mg/kg)<br><br>Pyrethroids: Cypermethrin (0.370 mg/kg), permethrin (0.217 mg/kg), deltamethrin (0.0152 mg/kg)<br><br>Organophosphates: Chlorpyrifos (0.00111 mg/kg)<br><br>Macrocyclic lactones: Ivermectin (0.360 mg/kg) | *major)* and Blue Tit (*Cyanistes caeruleus*) | used for lining nests in birds located near rural land. | and number of unhatched eggs positively correlated to increasing overall pesticide concentration in both species. Fibronil and imidacloprid concentration separately also positively correlated with the number of dead offspring only in the Great Tit. | Montaigu et al., 2025) |
| Reported as concentrations in pond water.<br><br>Organophosphates: dimethoate (0.000038 mg/L), chlorpyrifos-methyl (0.000020 mg/L)<br><br>Triazole Fungicides: Myclobutanil (0.000134 | Common toad (*Bufo bufo*) | Ponds located near agricultural areas, assumed water run-off contamination | Toads from ponds with higher pesticide concentrations laid more eggs but had decreased fertilization rates, as well as lower offspring survival rates and reduced | (Adams et al., 2021) |

| | | | | |
|---|---|---|---|---|
| mg/L), tebuconazole (0.000073 mg/L), penconazole (0.000047 mg/L), cyflufenamid (0.000020 mg/L), pyrimethanil (0.000028 mg/L)<br><br>Strobilurin Fungicides: Azoxystrobin (0.000043 mg/L), kresoxim-methyl (0.000220 mg/L), metrafenone (0.000141 mg/L)<br><br>Phenylpyrrole Fungicides: Fludioxonil (0.000084 mg/L)<br><br>Anilinopyrimidine Fungicides: Iprovalicarb (0.000176 mg/L)<br><br>Other fungicides: boscalid (0.000095 mg/L), folpet (0.000536 mg/L), fluopyram (0.000165 mg/L), fluopicolide (0.000061 mg/L), dimethomorph (0.000080 mg/L), famoxadone (0.000088 mg/L), metalaxyl-m (0.000160 mg/L), zoxamide (0.000080 mg/L) | | | offspring size during Gosner stage 25 | |
| Reported as concentrations in eggs of exposed species.<br>Neonicotinoids: Imidacloprid (358 ng/g lw), desnitro-imidacloprid (12.5 ng/g lw), imidacloprid-olefin (11.5 ng/g lw), | Black kite (*Milvus migrans*), Booted eagle (*Hieraaetus pennatus),* | Within eggs of exposed species, assumed exposure via parental ingestion/inhalation | Higher pesticide levels were found along decreased reproductive performance of booted eagles (lowered hatching success, breeding | (Peris et al., 2023) |

| | | | |
|---|---|---|---|
| acetamiprid (1.47 ng/g lw), desmethyl-acetamiprid (0.06 ng/g lw), thiacloprid (0.44 ng/g lw), thiamethoxam (0.34 ng/g lw), dinotefuran (7198 ng/g lw)<br><br>Pyrethroids: Cypermethrin (370 ng/g lw), permethrin (217 ng/g lw), deltamethrin (15.2 ng/g lw)<br><br>Organophosphates: Chlorpyrifos (1.11 ng/g lw)<br><br>Macrocyclic lactones: Ivermectin (360 ng/g lw) | Purple heron (*Ardea purpurea*), Black-headed gull (*Chroicocephalus ridibundus*), Slender-billed gull (*Chroicocephalus genei*), Gull-billed tern (*Gelochelidon nilotica*) | | success) |
